# Canopy temperature depression for drought- and heat stress tolerance in wheat breeding

**DOI:** 10.18699/VJGB-22-24

**Published:** 2022-03

**Authors:** S.B. Lepekhov

**Affiliations:** Federal Altai Scientific Centre of Agro-BioTechnologies, Barnaul, Russia

**Keywords:** CTD, wheat, drought tolerance, heat tolerance, selection criteria, показатель CTD, пшеница, засухоустойчивость, жаростойкость, критерий отбора

## Abstract

An infrared thermometer was first used to assess drought and heat tolerance in plant breeding more than 40 years ago. Soon afterward, this method became widely used throughout the world. However, Russia has not yet applied the described method for evaluating stress tolerance. This paper presents an overview of using infrared thermometry in plant breeding. Taking wheat as an example, it shows major advantages and disadvantages of canopy temperature depression (CTD) values measured by the infrared thermometer. The paper also demonstrates that genotypes with higher CTD values, and therefore with a lower canopy temperature, use more available soil moisture under drought stress to cool the canopy by transpiration. It refers to CTD as an integrative trait that reflects an overall plant water status. Its coefficient of variation lies in the interval of 10 to 43 %. A large number of publications illustrate a close relation between CTD values and yield and indicate a high heritability of the former. Meanwhile, the same works show that yield has a higher heritability. Moreover, some researchers doubt that CTD should be used in applied wheat breeding as there are many factors that influence it. CTD has a high correlation with other traits that reflect plant water status or their adaptation to drought or heat stress. Quantitative trait loci (QTLs) associated with CTD are localized in all chromosomes, except for 3D. These QTLs often explain a small part of phenotypic variance (10–20 %, more likely less than 10 %), which complicates the pyramiding of canopy temperature genes through marker-assisted selection. The paper concludes that the evaluation of CTD appears to be a reliable, relatively simple, labor-saving, objective, and non-invasive method that sets it apart from other methods as well as shows the best results under terminal drought and heat stress conditions

## Abstract (ru)

Прошло более 40 лет с начала использования инфракрасного термометра для оценки засухо- и жаростойкости в селекции растений. За это время метод широко распространился во всем мире. Однако в России описываемый способ оценки стрессоустойчивости сортов до сих пор не применяется. Нами сделан обзор результатов использования инфракрасного термометра в селекции растений. На примере пшеницы описаны основные достоинства и недостатки показателя CTD (сanopy temperature depression), оцениваемого посредством инфракрасного термометра. Генотипы с более высоким значением CTD, а значит, более прохладным пологом в условиях засухи, используют большее количество доступной почвенной влаги для охлаждения за счет транспирации. CTD – интегрирующий признак, который диагностирует текущий водный статус растений. Коэффициент вариации показателя CTD находится в пределах 10–43 %. В значительном количестве работ показана его тесная взаимосвязь с урожайностью и высокая наследуемость, однако в целом больший коэффициент наследуемости имела урожайность. Применение показателя CTD в практической селекции пшеницы оспаривается рядом исследователей из-за значительного количества влияющих на него факторов. CTD тесно связан с другими признаками, отражающими водный статус растений или результат адаптации к засухе или жаре. Локусы количественных признаков, ассоциированные с CTD, обнаружены на всех хромосомах, за исключением хромосомы 3D. Выявленные локусы часто описывают небольшую часть фенотипической изменчивости (10–20 %, чаще менее 10 %), что затруднит пирамидирование генов, связанных с температурой полога, посредством маркерной селекции. Оценка показателя CTD надежна, технически проста и производительна и при надлежащем ее использовании позволяет объективно определить одну из сторон жаро- и засухоустойчивости сортов, сохранив растения в живом виде, что выгодно отличает ее от других методов. Наилучший результат описываемый метод демонстрирует в условиях терминальной засухи.

## Introduction

Every year 200 million hectares of wheat (Triticum aestivum L.) cultivated worldwide suffer economic losses from drought and heat (Ortiz et al., 2008) for wheat is very sensitive to heat stress. Optimal temperature for photosynthesis in wheat is approximately 25 °C (Nagai, Makino, 2009). It has been estimated that 1 °C increase above the optimal temperature at the grain filling stage decreases wheat yield by 3–4 % (Wardlaw et al., 1989).

Breeding drought-tolerant cultivars is one of the possible ways to reduce damage from drought. However, it requires much time and effort as it includes the evaluation of a large number of plants and is complicated by a low and inconsistent correlation between the phenotype and the yield under drought conditions, with multiple mechanisms of adaptation being involved. The selection that is based solely on yield indicators complicates breeding for drought tolerance because the yield shows low heritability under drought stress (Sofi et al., 2019). With that in mind, to evaluate many genotypes in a short period of time, it is important to single out other traits associated with drought tolerance (Sohail et al., 2020).

The paper suggests several physiological traits to identify tolerant genotypes. It refers to physiological traits as traits that contribute to mechanisms playing a role in plant adaptation to stress (Reynolds et al., 2009), such as coleoptile length, ability to stay green, stem water soluble carbohydrate, leaf water potential, canopy temperature, and so on.

This paper presents an overview of using infrared thermometry in plant breeding.

## CTD and method of its measuring

Canopy temperature is an integrative trait that reflects the plant water status or the resultant equilibrium between the root water uptake and shoot transpiration (Berger et al., 2010). Under the high solar radiation and drought conditions, stomatal conductance decreases, soil moisture deficit reduces normal transpiration rate, which in turn increases canopy temperature (Rebetzke et al., 2013). Thus, canopy temperature can be used to study drought and heat tolerance in plants. Instead of canopy temperature, researchers often calculate canopy temperature depression (CTD) that refers to a metric, indicating the difference between air temperature and canopy temperature (Jackson et al., 1981). If the canopy temperature is lower than the air temperature under the influence of transpiration, then CTD is expressed as a positive value, but becomes negative if the reverse is true. Genotypes with higher CTD values and a cooler canopy temperature under drought stress use more available soil moisture to cool the canopy by transpiration. Given that Russian research lacks an established definition for ‘canopy temperature depression’, the paper refers to it as CTD defined in English research.

Canopy temperature is measured by a handheld infrared thermometer or thermal camera (Yousfi et al., 2019). It is done in the afternoon in clear weather conditions on windless days. The most considerable genotypic differences in CTD are reported from 2 to 3 p. m. (Thapa et al., 2018) at high temperatures and low relative humidity (Zhang X. et al., 2018). The researcher should stay close to the plot not to cast shadow on the place of measurement (Pinto et al., 2010). If a plot is sown in rows, it is best to stand to one side of it so that the infrared thermometer is pointed at an angle to the rows. If ground cover is low, it is best to point the thermometer at a low angle to the horizontal to minimize the likelihood of viewing soil (Reynolds et al., 2001). The infrared thermometer is held at approximately 50 cm above the canopy, and the measurements are taken at 1 m from the edge of the plot (Mason et al., 2011; Sohail et al., 2020). The best phase to perform measurements is the grain filling period (Thapa et al., 2018).

The infrared thermometer was first used for scheduling crop irrigation in the 1970s (Jackson et al., 1977) and for studying drought tolerance in the 1980s (Blum et al., 1982). In late 1980s, CIMMYT began to use CTD measurements as selection criteria in breeding for drought and heat tolerance in various experiments. Bulks showing high CTD values are selected in F3 generation (Blum, 2005). Canopy temperature measurements can significantly improve the selection of drought tolerant genotypes because of their high speed (≈10 seconds per plot), simplicity, and relative economic efficiency. CTD is also integrative of the whole canopy due to scoring many plants at once, thus reducing error associated with plant-toplant variation (Cossani, Reynolds, 2012).

## Factors inf luenced on the measuring accuracy of CTD

However, this method has some limitations. First, the measuring accuracy depends on microclimate of the plant stand. Second, rapid changes in environmental conditions, for example on cloudy days, demonstrate high variability of the results (Chaves, 2013). Third, CTD is influenced by many biological and environmental factors, such as air temperature and relative humidity, soil moisture, wind, solar radiation, evapotranspiration, leaf adjustment to water deficit (Bahar et al., 2008), plant density (White et al., 2012), spike size, color and size of leaves (Balota et al., 2008), angle of leaves (Zhang Y. et al., 2011), peduncle length and awns (Bonari et al., 2020). Finally, plant organs differ in their self-cooling abilities, and thus, canopy temperature with spikes is 2 °C higher than the one without them (Olivares-Villegas et al., 2007).

The fact that these limitations have already been identified allows us to conclude that CTD and its features are well researched. Some environmental flux during the measurement period is inevitable, but correcting data against reference plots, use of replication, and repetition of data collection during the crop cycle can compensate for this (Reynolds et al., 2001).

## Association of CTD with other traits of wheat

CTD values demonstrate a significant correlation with yield under drought and heat stress in a large number of experiments (Gao et al., 2016; Liang et al., 2018; Sohail et al., 2020). They have regression relationships: if CTD decreases by 1 °C, the yield declines by 1.5 and 1.7 q/ha (Kaur et al., 2018). In this regard, the trait should be considered as a significant selection criterion in breeding programs not only in Mexico, but also in other countries of the world (Al-Ghzawi et al., 2018; Thapa et al., 2018). Newer cultivars of wheat have cooler canopy (Thapa et al., 2018), although the cultivars that are released in different decades under favorable growing conditions or irrigation do not show this correlation (Balota et al., 2017).

Various studies identify high correlation between CTD and other traits that reflect plant water status or their adaptation to drought or heat, including stomatal conductance (Bonari et al., 2020), delay in the senescence of leaves (Fang et al., 2017), leaf and stem wax (Mondal et al., 2015), depth and distribution of root system in soil (Pinto, Reynolds, 2015), spike sterility (Sohail et al., 2020), and 1000 grain weight (Gulnaz et al., 2019).

## Variation and heritability of CTD

The coefficient of variation of CTD in different studies ranges from middle (10–14 % (Sharma P. et al., 2017; Jokar et al., 2018)) to high (26–43 % (Kumar et al., 2017; Sharma D. et al., 2018)). Dryland conditions make CTD values negative (Thapa et al., 2018) and increase genotypic differences (Pinto et al., 2010). In this respect, CTD value appears to be a better parameter for drought tolerance than yield under drought stress. Some research suggests that canopy temperature has a larger genetic value – if compared to direct selection based on yield and other traits – as it is an indirect index to the selection of certain types of cultivars and shows higher heritability and genetic correlation with yield (Rebetzke et al., 2013). Although some studies put CTD heritability at 0.65–0.80 (Kumar et al., 2017; Khan et al., 2020), there is a vast amount of research that calculates heritability for both CTD and yield and concludes that the latter has the larger heritability (see the Table).

**Table 1.**
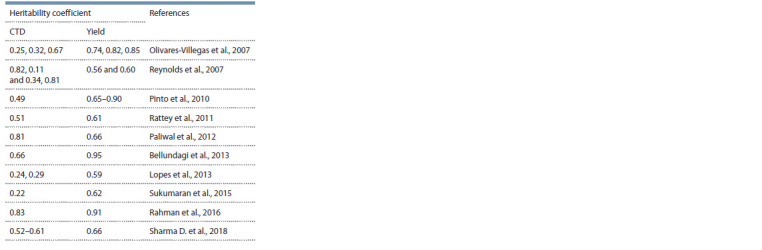
Heritability coeff icient for CTD and yield of common wheat in different studies

One of the possible reasons for the low heritability of CTD value is environmental influence (Gao et al., 2016). Thus, literature review demonstrates that CTD cannot be referred to as the better selection criteria if compared to yield criteria under drought stress. This indicator is better used as an additional parameter in measuring drought tolerance in cultivars.

## Genetic basis for canopy temperature depression

CTD genetic control has been extensively studied during the last two decades. For example, in their study, Acuña-Galindo et al. (2015) analyzed 30 pieces of research from 2002 to 2011 and identified four meta-QTLs (MQTLs) containing two or more QTLs for the trait that were associated with drought and heat tolerance, including CTD value that was identified in independent studies, populations, or environments. These MQTLs were localized on chromosomes 1B (34 ± 2 сМ), 2B (68 ± 2 сМ), 3B (139 ± 4 сМ), and 7A (100 ± 6 сМ). The research also described single QTLs for CTD: these MQTLs were localized on chromosomes 3B, 4A, 7A, while chromosome 5A contained three MQTLs. QTLs associated with CTD were co-localized with QTLs that controlled other adaptive traits (yield, biomass, days to heading, grains per spike, 1000 grain weight, and water-soluble carbohydrates). While summarizing the results of their and prior research, Pinto et al. (2010) suggested that QTLs for canopy temperature were localized on chromosomes 1A, 1B, 1D, 2A, 2B, 3A, 3B, 4A, 4B, 5A, 5B, 6A, 6B, 6D, 7A, and 7B. The research undertaken after 2011 discovered QTLs for CTD on almost all chromosomes, except for 1D, 3A, 3D, and 6D (Paliwal et al., 2012; Lopes et al., 2013; Mason et al., 2013; Rebetzke et al., 2013; Mondal et al., 2015; Sukumaran et al., 2015; Awlachew et al., 2016; Gao et al., 2016; Mohammed et al., 2021).

Some research showed that QTLs associated with CTD were co-localized or closely localized with genes Rht-B1 (Gao et al., 2016), Rht-D1 (semi-dwarf wheat with warmer canopy), Ppd-D1 (Rebetzke et al., 2013), Vrn-A1 (Mondal et al., 2015), and transcription factor Dreb1 (Khalid et al., 2019).

These loci for canopy temperature are responsible for 10– 20 % of the phenotypic variation (Paliwal et al., 2012; Mondal et al., 2015; Awlachew et al., 2016) or even less than 10 % (Rebetzke et al., 2013; Sukumaran et al., 2015), and this is understandable as CTD is an integrative trait that is correlated to many mechanisms of drought tolerance (Lopes et al., 2013). Moreover, canopy cooling at different stages is controlled by loci with different localization (Lopes et al., 2013; Gao et al., 2016); therefore, the result of CTD measurements depends on the plant growth stage (Gulnaz et al., 2019). It is likely that the small genetic effects of multiple QTLs combined with the smaller population sizes commonly used in breeding will limit the pyramiding of multiple alleles for CTD through markerassisted selection (Rebetzke et al., 2013).

## Problems of using CTD in applied breeding

CTD indicates any kind of stress: high temperature, water or nutrient shortage (Kaur et al., 2018). Nitrogen fertilizers increase CTD values (Yang et al., 2018). Thus, this parameter shows not only water, but also a nitrogen nutrient status of plants (Guo et al., 2016). CTD is also related to NDVI (Yousfi et al., 2019), and canopy temperature may increase due to Zymoseptoria tritici infection (Wang et al., 2019). At the same time, high canopy temperatures provide unfavorable conditions for the development of stripe rust (Cheng et al., 2015). As the environmental factors have the aggregate effect on plants, CTD measurements that are performed for screening of drought tolerance without drought stress may produce incorrect results.

In addition to the well-studied negative correlation between yield and CTD values under drought or heat stress, the researchers highlight the controversy of their relationships in various environments (Balota et al., 2017). For example, in a high-yielding environment, cultivars with relatively high CTD values tend to produce higher yields than those with low CTD values, while in a low-yielding environment, the relationship between these traits disappears (Lu et al., 2020). However, it is explained by the fact that differences in plant tolerance become noticeable only if limiting factors are intense (Udovenko, 1973). Some studies show insignificant or positive correlation between CTD and yield (Rahman et al., 2016; Bala, Sikder, 2017), while others identify that under drought stress, high-yielding genotypes have both positive and negative CTD values (Sofi et al., 2019).

The research shows that a relatively large proportion of yield phenotypic variation under drought stress can be explained by a small number of traits, including CTD values. In most cases, they would be amenable to reliable quantification in parents and verification of expression in segregating progeny (Reynolds et al., 2007). However, it was impossible to accurately measure CTD values in some research because the plant canopy failed to cover the ground (Liang et al., 2018) or the yield was highly dependent on limited amounts of soilstored water (Royo et al., 2002). Thus, Balota et al. (2017) identified the difficulty of using CTD values in applied plant breeding, for in that work a potential effect of neighbor plots plant height on canopy temperature was present.

More than that, a certain amount of caution is advisable in selecting genotypes with high CTD values in water-limited environments as more vigorous, later-flowering wheats may produce more biomass by the time canopy temperature is measured. Biomass and transpiration are physiologically linked, so that higher-biomass lines seem to deplete soil water faster, causing stomata to close and canopies to warm. Selection of cooler canopy temperature under conditions of soil-water depletion could favor the development of lines with low yield potential and smaller biomass (Rebetzke et al., 2013) or identification of specific genotypes (Jokar et al., 2018). a non-invasive method, and this sets it apart from others. To better evaluate cultivars tolerance to drought or heat stress, a substantial number of traits should be considered, therefore making CTD a meaningful contribution to knowledge on drought tolerance. Still, it is important to realize that this method shows the best results under terminal drought and heat stress conditions.

## Conclusion

All things considered, the paper suggests that in general the evaluation of CTD appears to be a reliable, relatively simple, labor saving, and objective method that may be used to assess plant tolerance to heat or drought stress. Moreover, it is a non-invasive method, and this sets it apart from others. To better evaluate cultivars tolerance to drought or heat stress, a substantial number of traits should be considered, therefore making CTD a meaningful contribution to knowledge on drought tolerance. Still, it is important to realize that this method shows the best results under terminal drought and heat stress conditions.

## Conflict of interest

The authors declare no conflict of interest.
